# Supplementary material for: High-resolution CTCF footprinting reveals impact of chromatin state on cohesin extrusion dynamics
Source: bioRxiv. 2023 Oct 28:2023.10.20.563340. Preprint. [Version 3] doi: 10.1101/2023.10.20.563340 (PMC10634716; doi:10.1101/2023.10.20.563340)
Supplement: 1 [file NIHPP2023.10.20.563340V3-supplement-1.pdf]

bioRxiv preprint doi: https://doi.org/10.1101/2023.10.20.563340; this version posted October 28, 2023. The copyright holder for this preprint (which was not certified by peer review) is the author/funder, who has granted bioRxiv a license to display the preprint in perpetuity. It is made available under aCC-BY 4.0 International license.

Supplementary Figures

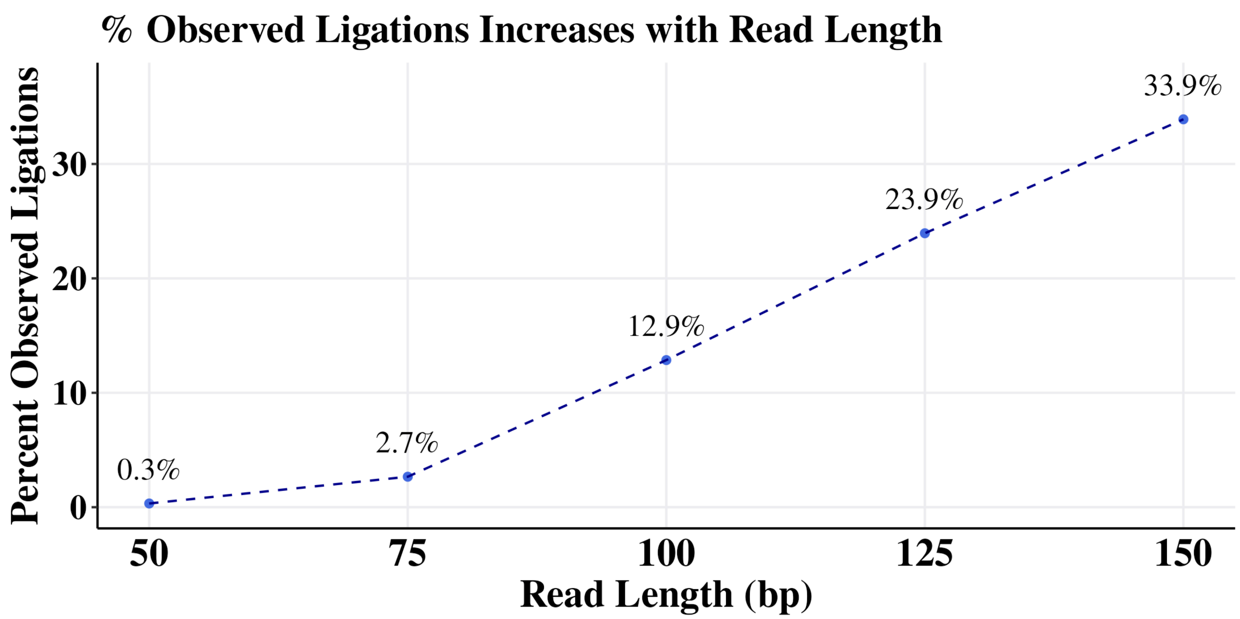

Supplementary Figure 1. Percent observed ligations increases with read length.

bioRxiv preprint doi: https://doi.org/10.1101/2023.10.20.563340; this version posted October 28, 2023. The copyright holder for this preprint (which was not certified by peer review) is the author/funder, who has granted bioRxiv a license to display the preprint in perpetuity. It is made available under aCC-BY 4.0 International license.

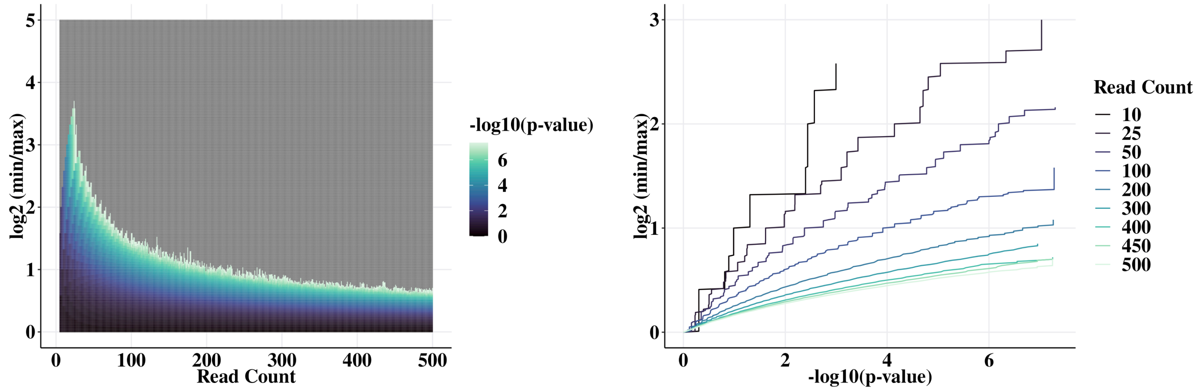

Supplementary Figure 2. The probability of observing a high *FactorFinder* statistic under the null hypothesis is higher at low read counts.

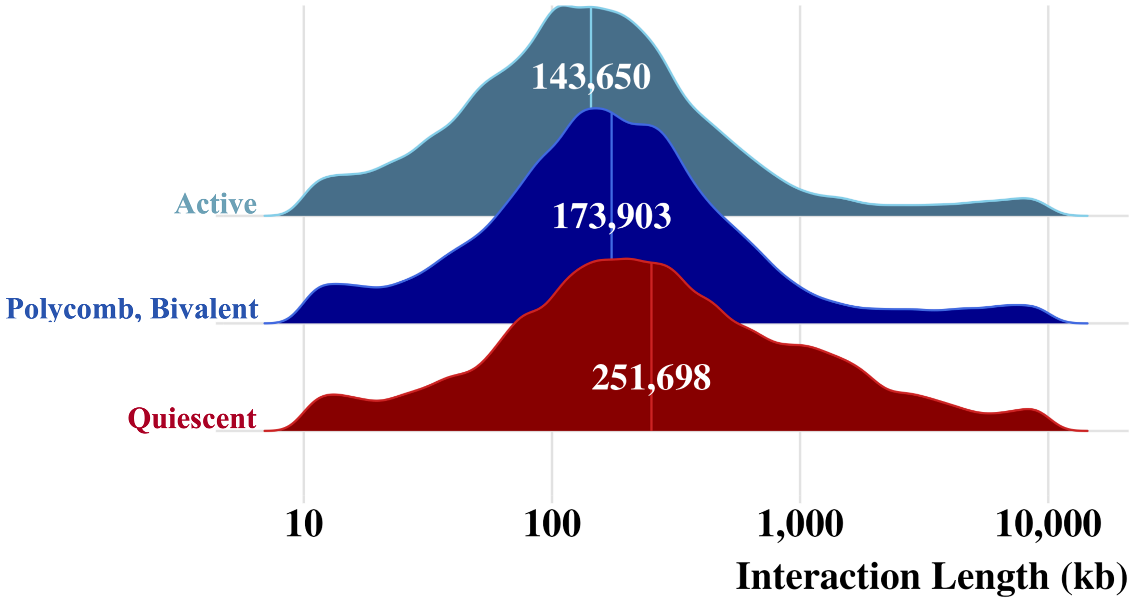

Supplementary Figure 3. Cohesin extrudes significantly further through quiescent regions than active regions.

bioRxiv preprint doi: https://doi.org/10.1101/2023.10.20.563340; this version posted October 28, 2023. The copyright holder for this preprint (which was not certified by peer review) is the author/funder, who has granted bioRxiv a license to display the preprint in perpetuity. It is made available under aCC-BY 4.0 International license.

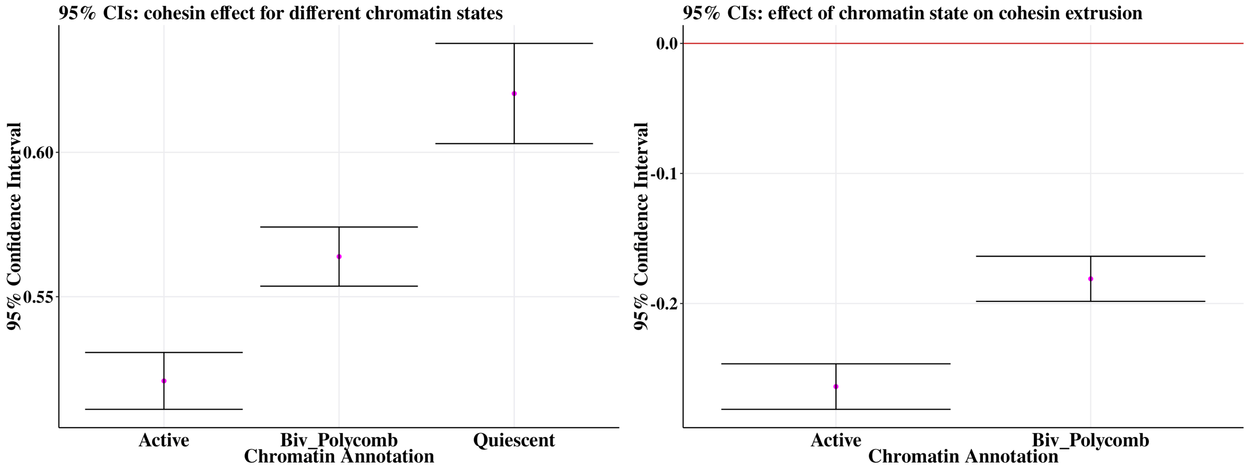

Supplementary Figure 4. Controlling for locus-specific variation with linear mixed models does not attenuate the relationship between chromatin state and extruded loop size. Note that for the figure on the right, the group that active and bivalent polycomb are being compared to is quiescent.

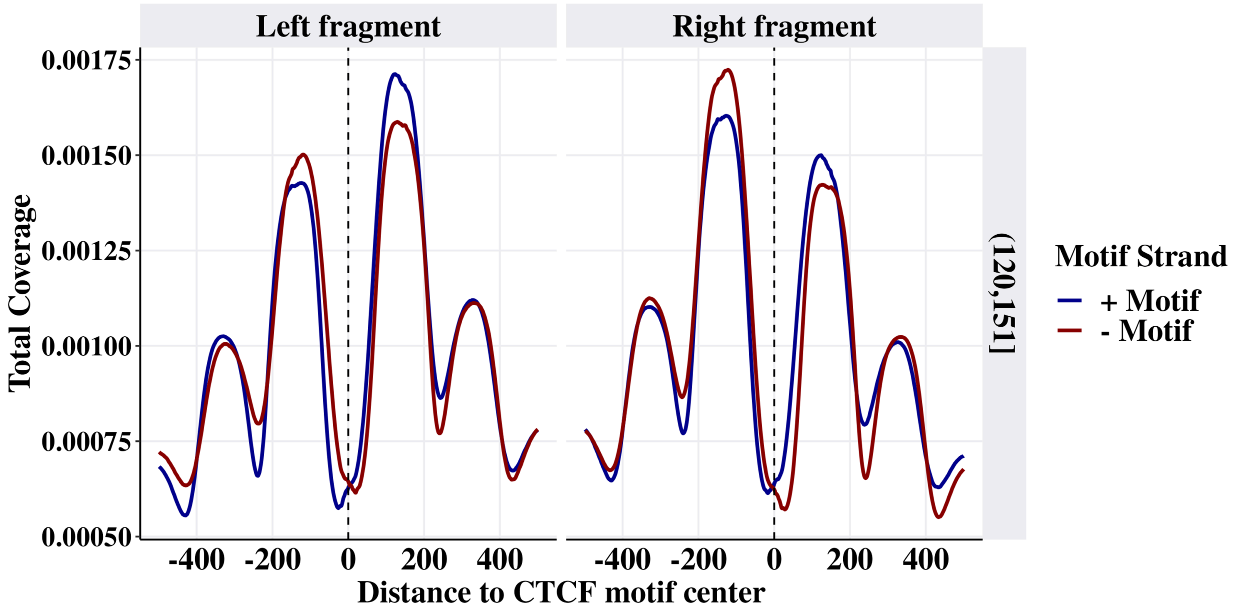

Supplementary Figure 5. Nucleosomes are preferentially positioned inside the loop.
